# Assessing the Synergistic Activity of Clarithromycin and Therapeutic Oils Encapsulated in Sodium Alginate Based Floating Microbeads

**DOI:** 10.3390/microorganisms10061171

**Published:** 2022-06-07

**Authors:** Ikram Ullah Khan, Mehwish Shoukat, Muhammad Asif, Syed Haroon Khalid, Sajid Asghar, Muhammad Usman Munir, Muhammad Irfan, Akhtar Rasul, Sameer H. Qari, Alaa T. Qumsani, Mohamed M. Hassan, Maryam A. Alahdal, Muhammad Usman, Zulqurnain Khan

**Affiliations:** 1 Department of Pharmaceutics, Faculty of Pharmaceutical Sciences, Government College University Faisalabad, Faisalabad 38000, Pakistan; mesh.786@hotmail.com (M.S.); haroonkhalid80@gmail.com (S.H.K.); sajuhappa@gmail.com (S.A.); manipharma1@gmail.com (M.I.); dr.akhtarstar@gmail.com (A.R.); 2 Department of Pharmacology, Faculty of Pharmacy, The Islamia University of Bahawalpur, Bahawalpur 63100, Pakistan; asif_pharmacist45@yahoo.com; 3 Department of Pharmaceutical Chemistry, College of Pharmacy, Jouf University Sakaka, Sakakah 72388, Saudi Arabia; mumunir@ju.edu.sa; 4 Department of Biology, Al-Jumum University College, Umm Al-Qura University, Makkah 24382, Saudi Arabia; shqari@uqu.edu.sa (S.H.Q.); atqumsani@uqu.edu.sa (A.T.Q.); 5 Department of Biology, College of Science, Taif University, Taif 21944, Saudi Arabia; m.khyate@tu.edu.sa; 6 Biology Department, Faculty of Applied Sciences, Umm Al-Qura University, Makkah 21514, Saudi Arabia; mayahdal@uqu.edu.sa; 7 Department of Biochemistry and Biotechnology, MNS University of Agriculture, Multan 66000, Pakistan; muhammad.usman@mnsuam.edu.pk; 8 Institute of Plant Breeding and Biotechnology (IPBB), MNS University of Agriculture, Multan 66000, Pakistan; zulqurnain.khan@mnsuam.edu.pk

**Keywords:** floating microbeads, clarithromycin, eucalyptus oil, oleic acid, sodium alginate

## Abstract

We developed alginate-based floating microbeads of clarithromycin with therapeutic oils for the possible eradication of *Helicobacter pylori* (*H. pylori*) infections by enhancing the residence time of the carrier at the site of infection. In pursuit of this endeavor, the alginate was blended with hydroxy propyl methyl cellulose (HPMC) as an interpenetrating polymer to develop beads by ionotropic gelation using calcium carbonate as a gas generating agent. The developed microbeads remained buoyant under gastric conditions for 24 h. These microbeads initially swelled and afterwards decreased in size, possibly due to the erosion of the polymer. Furthermore, swelling was also affected by the type of encapsulated oil, i.e., swelling decreased with increasing concentrations of eucalyptus oil and increased with increasing concentrations of oleic acid. Antibacterial assays of the formulations showed significant antibacterial activity against *Staphylococcus aureus* (*S. aureus*) and *Escherichia coli* (*E. coli*); these assays also showed synergistic activity between clarithromycin and therapeutic oils as evident from the higher zone of inhibition of the microbeads as compared to the pure drug and oils. Scanning electron microscopy (SEM) images revealed a smoother surface for oleic acid containing the formulation as compared to eucalyptus oil containing the formulation. Differential scanning calorimetry (DSC) and thermogravimetric analysis (TGA) revealed the development of a stable formulation, while Fourier transform infrared spectrophotometry (FTIR) studies did not reveal any interaction between the polymers and the active ingredients. Optimized formulations (CLM3 and CLM6) were designed to release the drug in a controlled manner in gastric media by Fickian diffusion. Conclusively, the developed microbeads are a promising carrier to overcome the narrow therapeutic index and low bioavailability of clarithromycin, while the presence of therapeutic oils will produce synergistic effects with the drug to eradicate infection effectively.

## 1. Introduction

Pharmaceutical scientists are continuously striving to achieve improved drug bioavailability after oral administration, as it is the most widely used and convenient route of administration. Conventional oral drug delivery systems are not able to overcome all the difficulties imposed by the gastrointestinal tract due to the various physiological constraints arising from the heterogeneity of the gastrointestinal tract. Various major obstacles faced during oral drug delivery include variable gastric emptying patterns, minimum drug residence, and negligible drug release from the carrier in a specific target area [1]. In recent times, gastroretentive drug delivery systems (GRDDS) have emerged as a promising approach to improve the bioavailability and effectiveness of active pharmaceutical ingredients (API) with an absorption window in the stomach or upper small intestine, or to promote localized effects in the stomach and duodenum [2,3]. GRDDS not only enhance the bioavailability, but also lower the required dose and minimize the gastrointestinal side effects. Candidates suitable for gastroretentive drug delivery systems include (a) drugs with a narrow therapeutic window in the stomach and small intestine, (b) drugs with a short half-life, (c) drugs that undergo instability and degradability in the lower gastrointestinal tract environment, (d) drugs that exhibit local activity in the upper part of the gastrointestinal tract (GIT), and (e) drugs with a low solubility at alkaline pH [4].

In GRDDS, various factors affect the gastric retention of the dosage forms including pharmaceutical technology factors (density of dosage form, size of dosage form), extrinsic factors (nature, posture, physical activity, sleep, frequency of food ingestion, body mass index, caloric content, and co-administration of drugs that affect the motility pattern) [5,6], and biological factors (age, illness, gender, and emotional state) [6,7]. The literature mentions several approaches to enhance the gastric residence time including muco- or bio-adhesive systems, expandable systems, high-density systems, floating systems, magnetic systems, and superporous hydrogels [3].

Among the various GRDDS described in the literature, floating drug delivery systems (FDDS) are the most prominent. FDDS hold the capacity to float in and over the gastric contents owing to their low density and must be below 1.004 g/cm^3^ [2,8]. FDDS are more suitable for localized treatments, such as those of local infections, peptic ulcers, gastritis, and Zollinger–Ellision syndrome, and are equally applicable for systemic applications. FDDS also provide protection to acid-labile and short half-life drugs. Furthermore, these systems are known to reduce the wastage of drugs and improve bioavailability by enhancing the residence time of drugs in the absorption window region of the GIT [8,9].

Clarithromycin (CLM) belongs to the macrolide class and inhibits bacterial protein synthesis. It belongs to BCS class II with a low solubility and high permeability. Its half-life is 3–4 h, and its bioavailability is 50% [10]. However, it is better absorbed from the stomach and also remains stable under acid conditions [11]. *H. pylori* resistance to various antibiotics including CLM is continuously rising [12,13], and thus there is need to develop new pharmaceutical strategies or carriers to overcome this resistance. This makes *H. pylori* infection a suitable candidate for GRDDS.

Low-density systems, such as FDDS, float on the stomach contents for a prolonged time while continuously releasing encapsulated contents. The composition of FDDS plays a key role in imparting floating characteristics and could comprise entrapped air or oil [14]. In the gas-generating system, various gas-generating agents such as calcium carbonate, sodium bicarbonate, tartaric acid, and citric acid are used. These gas-generating agents, upon contact with the gastric contents, produce carbon dioxide which is trapped in dosage form and keeps them buoyant in the gastric contents for a longer period and provides the slow release of the drug contents in the stomach [15]. Oils are trapped in carriers to impart a low density. Here, we develop microbeads with gas-generating agents, eucalyptus oil, and oleic acid. These oils may play multiple roles, i.e., (a) providing antibacterial activity against Gram-positive as well as Gram-negative bacteria [16] and (b) giving synergistic effects with clarithromycin. (c) Oil acts as a floating aid by creating multiple tiny pockets in the matrix to enhance buoyancy, and furthermore (d) helps to incorporate hydrophobic clarithromycin in microbeads. Thus, the developed floating microbeads containing clarithromycin and therapeutic oils will be a promising carrier for the treatment of *H. pylori*.

## 2. Material and Methods

### 2.1. Materials

Clarithromycin was received as a kind gift from Saffron Pharmaceutical Pvt (Ltd.), Faisalabad, Pakistan. Sodium alginate (CAS No 9005-38-3), hydroxy propyl methyl cellulose (HPMC) K15M (Viscosity = 15,000 cps), and calcium chloride (CAS No 10043-52-4) were obtained from Daejung Chemical and Metals Co., Ltd., Sinan, Gyeonggi, Korea. Calcium carbonate (CAS No 471-34-1) and acetic acid (CAS No 64-19-7) were obtained from E Merck, Darmstadt, Germany. Essential oils (eucalyptus oil (CAS 8000-48-4), oleic acid (CAS 112-80-1)) were obtained from Sigma Aldrich (St. Louis, MO, USA).

### 2.2. Preparation of Microbeads

Clarithromycin-loaded floating microbeads were prepared by an ionotropic gelation method. Initially, a 0.5% *w*/*v* solution of sodium alginate was prepared in distilled water. Afterward, 0.25% *w*/*v* HPMC K15M was added into the sodium alginate solution and stirred continuously by a magnetic stirrer. In the next step, 0.5% *w*/*v* calcium carbonate was added into this solution by continuous stirring. Clarithromycin was added to different concentrations of oils (0.2% to 0.5% *w*/*v*) and stirred to obtain a clear dispersion (Table 1). Later, the drug and oil mixture was added to the above polymeric solution and stirred continuously for 15 min. The final solution was added dropwise into a mixture containing 2% *w*/*v* calcium chloride and 10% *v*/*v* acetic acid solution. The polymeric solution was introduced into the cross linker solution at a constant flow rate of 10 mL/min by a needle of suitable gauge, using a syringe pump (ENMIND). After 30 min of curing, the microbeads were filtered, washed with distilled water, and air dried [11].

### 2.3. Characterization

#### 2.3.1. Particle Size Analysis

The particle sizes of microbeads prepared with different gauge needles were determined using an ocular micrometer fitted in the eyepiece of an optical microscope and calibrated with a stage micrometer. Fifty microbeads were individually analyzed and the average reported [17].

#### 2.3.2. Swelling Study

The swelling of the beads was checked by immersing the microbeads in 100 mL of 0.1 N HCl (pH 1.2). Afterwards, the beads were removed, dried, and weighed at different time intervals (0.5, 1, 2, 3, and 4 h). The dynamic weight changes of the microbeads were calculated as follows:

% weight change = Ws − Wi/Wi × 100

where “Ws” is the weight of the beads in the swollen state, and “Wi” is the initial weight of the beads [11].

#### 2.3.3. In Vitro Buoyancy and Lag Time

The in vitro buoyancy was determined at different time intervals using fifty dry beads placed in 0.1 N HCL (pH 1.2) containing 0.02% *w*/*v* Tween 20. The temperature was maintained at 37 ± 1 °C and agitated at 100 rpm for 24 h using a USP Type II dissolution apparatus. The time taken by the beads to rise to the surface and float was taken as the lag time [11].

#### 2.3.4. Density Measurements

The density of the microbeads was measured by filling 10 mL of a volumetric cylinder to the mark. The bulk density (Pb) of the beads was calculated by using the formula:

Pb = (W2 − W1)/volume filled

where “W2” is the weight of the cylinder and microbeads, and “W1” is the weight of the empty cylinder [18].

#### 2.3.5. Drug Loading and Drug Entrapment Efficiency

A total of 25 mg of beads was placed in 20 mL of 0.1 N HCL and agitated at 100 rpm for 48 h. The drug loading and drug entrapment efficiency of all formulations were calculated by the following equations [11]:

Drug loading (% *w*/*w*) = amount of drug in sample (mg)/sample weight (mg) × 100

DEE (%) = % drug loading/% theoretical drug loading × 100

#### 2.3.6. Microscopic Studies

A trinocular microscope (Accu-Scope 3001) fitted with a 5 megapixel camera was used to capture optical images of microbeads in dry and swollen state. The surface morphology and shape were assessed using a scanning electron microscope (SEM). The dry samples of blank, CLM3, and CLM6 were placed on aluminum stubs and coated with gold before taking SEM images [19].

#### 2.3.7. Fourier Transform Infra-Red Spectroscopy (FTIR)

The FTIR spectra were recorded for the pure drug, blank, and drug-loaded microbeads to analyze the drug–polymer interactions. All samples were scanned in the range of 4000–400/cm [11].

#### 2.3.8. Thermal Analysis

Thermogravimetric analysis (TGA) was used to assess the weight changes in a given sample as a function of heat. Differential scanning calorimetry (DSC) analysis was performed to determine any physical changes given a material under test with an increasing temperature over the time. For analysis, 5 mg of each sample was heated in a temperature range of 25 °C to 300 °C at a rate of 10 °C/min under a nitrogen atmosphere [11].

#### 2.3.9. Powder X-ray Diffraction (XRD)

Powder X-ray diffraction was used for qualitative analysis of the crystalline and amorphous states of the pure drug and formulations. Pure drug, blank, CLM3, and CLM6 were scanned between 5° and 40° [11].

#### 2.3.10. Antibacterial Assay

Antibacterial activity was evaluated against the Gram-positive and Gram-negative bacterial strains, namely *Staphylococcus aureus* (ATCC 6538P) and *Escherichia coli* (ATCC 25922), respectively. The agar well diffusion method was used to assess the antibacterial activity of the pure drug, oil, and developed formulations. Briefly, nutrient agar media was prepared and incubated for 2 h, then solidified in petri plates under a laminar airflow hood. Afterwards, the surface of the solidified nutrient media was streaked with a standard inoculum of microorganism. Bacterial cultures were grown overnight and adjusted to McFarland standard 1, which was equivalent to 3.0 × 10^8^ CFU/mL of *S. aureus* and 3.5 × 10^8^ CFU/mL of *E coli*. Then, a 5–8 mm hole was punched in the solidified media with a sterile cork borer and filled with the standard drug, pure oil, blank, and formulations (CLM1 to CLM6). These plates were incubated at 37 °C for 24 h, and zones of inhibition were measured [20].

#### 2.3.11. In Vitro Drug Release and Release Kinetics

Drug release was assessed in a USP dissolution apparatus II using microbeads equivalent to 100 mg of drug. Each vessel contained 0.1 N HCl (pH 1.2) as a dissolution medium agitated at 100 rpm with the temperature maintained at 37 ± 1 °C. Samples (5 mL) were drawn at 0, 30, 60, 120, 180, 240, 300, 360, 420, 480, and 1440 min. Each sample was replaced with an equal volume of fresh dissolution media (0.1 N HCL). All samples were analyzed for drug content at 210 nm by using HPLC as described previously [21]. Release kinetics were assessed by the Korsemeyer–Peppas model [22].

## 3. Results and Discussion

### 3.1. Particle Size Analysis

**Figure 1:**
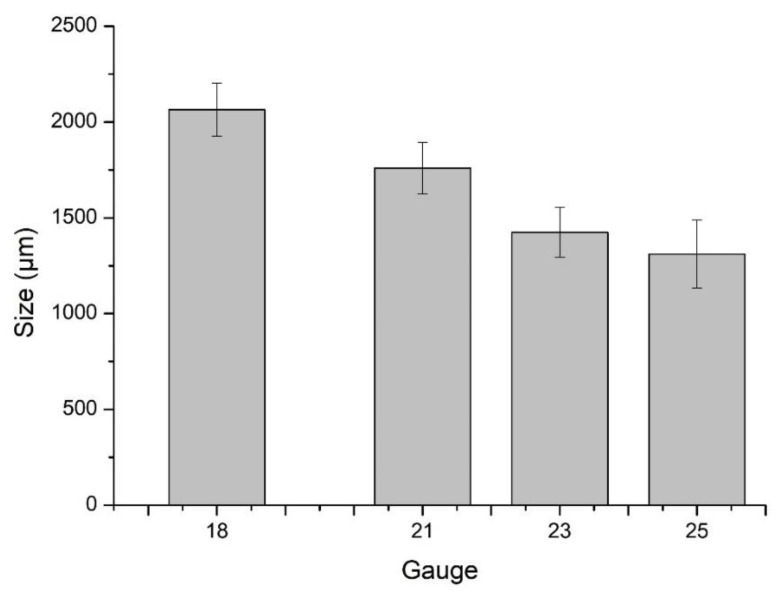
Microbead size variation with various syringe needle gauges. Error bars represent standard deviation (n = 50).

The particle size of microbeads is an important parameter which regulates the amount of active ingredient to be encapsulated and determines the possible route of administration. The particle size of microbeads can be controlled by the quantity of polymer, the flow rate of pump, and by variating the needle gauge. Here, we kept the first two factors constant and only varied the needle gauge diameter, i.e., 18, 21, 23, and 25. It is evident from Figure 1 that as the needle size becomes narrow, the bead size also decreases. Amsden and Goosen prepared calcium alginate microbeads and obtained variable size beads by changing the gauge of needle. Smaller microbeads were formed using a higher gauge needle. A higher gauge needle has a small internal diameter that leads to a smaller cross-sectional surface area from which the drop releases, and hence the size of microbeads is reduced [23]. In another study, alginate microbeads were developed in the range of 200–800 µm by varying the alginate concentration, flow rate, air pressure, and needle diameter. They obtained smaller beads with a smaller needle diameter [24]. For further analysis, microbeads were generated with a needle of 21 gauge. This gauge was selected as it produced optimum size microbeads and did not face difficulty in transferring the viscous polymer dispersion via the needle into the cross-linking solution.

### 3.2. Swelling Studies

**Figure 2:**
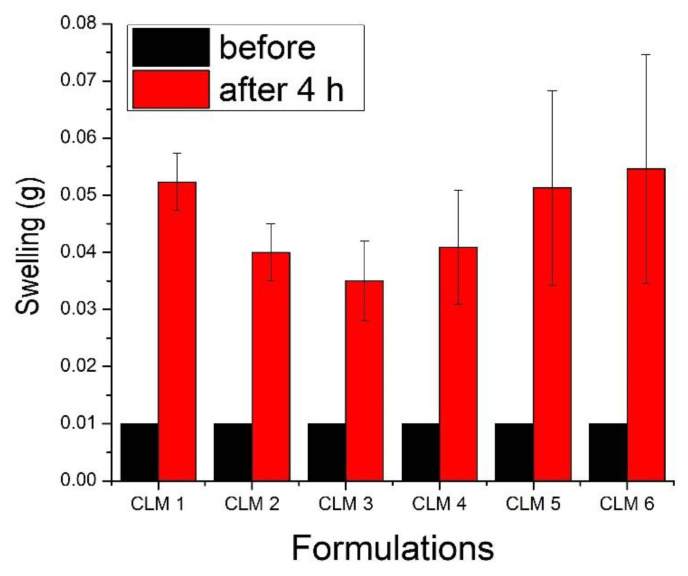
Swelling behavior of all formulations showing variation with respect to the concentration of oils. Error bars indicate standard deviation (n = 3).

The swelling of all formulations was tested in simulated gastrointestinal media for four hours to assess the impact of formulation ingredients on swelling characteristics as it is one of the key parameters influencing the drug release pattern from polymeric beads [25,26]. It is evident that the swelling of the first three formulations, i.e., CLM1, CLM2, and CLM3, decreased with increasing concentrations of eucalyptus oil. In contrast, the swelling of the next three formulations, i.e., CLM4, CLM5, and CLM6, increased with increasing concentrations of oleic acid (derived from olive oil) as shown in Figure 2. This is probably due to the structural differences of the two oils. Eucalyptus oil contains three (aromatic ring, alkyl, and ether groups) lipophilic groups in its structure [27]. As the concentration of eucalyptus oil increases, the concentration of lipophilic moieties is also increased, thus impeding the penetration of fluid in microbeads. Hence, the swelling decreased as the concentration of eucalyptus oil increased. Our results are in agreement with previous studies where the presence of hydrophobic oils reduced the swelling behavior of carriers by hindering the interaction of polymeric chains with dissolution media [28,29].

In contrary to this, as the concentration of oleic acid increased, it’s swelling also increased. Oleic acid is composed of long chain unsaturated fatty acid, which contains a hydrophilic group, i.e., carboxylic acid [30]. Thus, increasing the concentration of oleic acid, favors the penetration of fluid into the microbeads structure and hence increased swelling.

**Figure 3:**
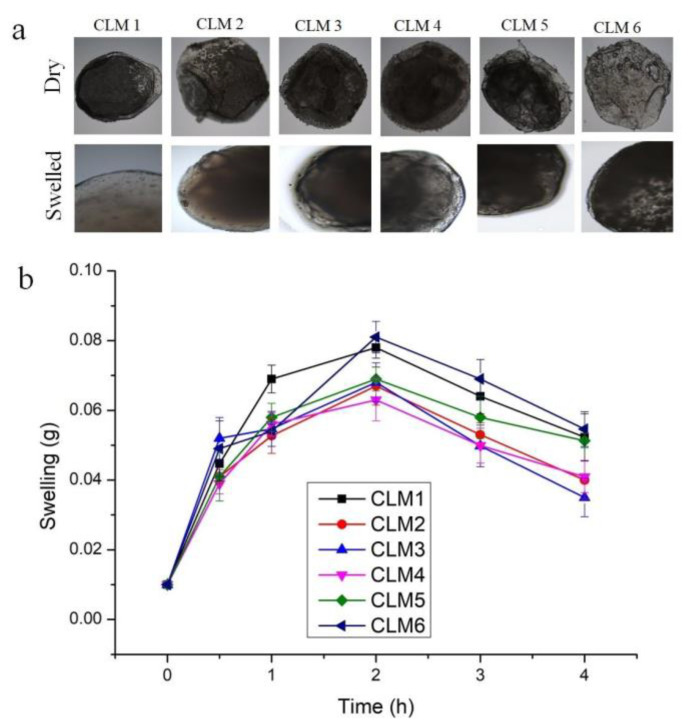
(**a**) Optical images of all formulations in dry and in swollen form after four hours in pH 1.2. (**b**) Swelling of all formulations at different time intervals. Error bars represent standard deviation (n = 3).

We also assessed the swelling of all formulations at different time intervals (i.e., 0 h, 0.5 h, 1 h, 2 h, 3 h, and 4 h) to evaluate the changes over the time. The results shows that initially weight of microbeads increased up to two hours and then it decreased (Figure 3).

Although, sodium alginate shows pH-dependent swelling, which is relatively higher at a higher pH owing to the presence of a –COOH group. At a low pH, this group remains partially unionized and develops hydrogen bonding between polymeric chains and solvent molecules, thus resulting in shrinkage and a low swelling profile at pH 1.2 [31]. The swelling of microbeads is also influenced by the presence of an interpenetrating polymer, i.e., HPMC K15, which hydrates rapidly upon contact with the dissolution media, thus leading to swelling of the microbeads. Similar observations have been reported for glimepiride-loaded microbeads developed with low-methoxy pectin and HPMC K15M. The presence of HPMC K15M imparts a rapid and higher swelling at pH 1.2 [32]. Maximum swelling was observed after 2 h, then it started to decrease—probably due to the erosion of the polymer as observed previously [33].

### 3.3. Buoyancy Studies

During buoyancy studies, all the microbeads started to float in less than one minute (Table 2) and continued to float for variable times. CLM1, CLM2, and CLM3 contain 0.3, 0.4, and 0.5% *w*/*v* of eucalyptus oil, respectively. As the concentration of oil was increased in the formulation, the in vitro buoyancy duration also increased. This is because oil not only imposes hydrophobic characteristics, but also aids in floating. Oil creates multiple tiny pockets in the polymer matrix to enhance buoyancy [11]. In CLM3, after 1 h 72% beads and after 4 h 60% beads were floating. At the end of 24 h, 6% beads were still floating, which shows excellent buoyancy (Table 2). A similar trend was observed with the formulations CLM4, CLM5, and CLM6 containing 0.2, 0.25, and 0.3% *w*/*v* oleic acid, respectively. As the concentration of oleic acid increased, the duration of floating of the microbeads also increased. In CLM6, which contains the maximum amount of oleic acid, after 1 h 66%, after 4 h 40%, and after 24 h only 4% beads were floating (Table 2). The slightly better floating duration of eucalyptus-oil-containing formulations was attributed to the higher concentration of oil as compared to the formulations containing oleic acid. In a previous study, Pornsak, Nartaya et al. assessed the floating behavior of oil-entrapped beads by using various types and concentrations of oils. They observed that as the concentration of oil was increased, its in vitro buoyancy also increased [34]. Clarithromycin has proven to be a very effective mono therapy for the treatment of *H. pylori*. However, its effectiveness is compromised due to the short dwelling time of clarithromycin in the stomach contents [35]. Increasing the residence time of the drug along with a co-agent could prove effective in the complete eradication of *H. pylori.*

### 3.4. Density Measurement

The results showed that CLM1, CLM2, and CLM3, which contain eucalyptus oil, have densities of less than one (Table 3). This reflects their suitability for floating drug delivery systems as this will aid in floating of the carriers. Similarly, the formulations containing oleic acid (CLM4, CLM5, and CLM6) have bulk densities of less than one, which promotes buoyancy (Table 3). Stops, Fell et al. determined the density of alginate-based microbeads. The calculated densities of microbeads were less than 1 g/mL. The results therefore suggest that alginate beads float when placed in aqueous media (0.1 NHCl) [36].

### 3.5. Drug Loading and Entrapment Efficiency

Table 3 shows the drug loading and entrapment efficiency of all formulations containing clarithromycin with eucalyptus oil or oleic acid. It was observed that the entrapment efficiency increased with increasing concentrations of eucalyptus oil. This is because CLM3 contains the maximum amount of eucalyptus oil with the highest contents of lipophilic groups to entrap the lipophilic drug. In the case of the oleic acid formulation, the entrapment efficiency increased with decreasing concentrations of oil as shown in Table 3. The probable reason for this trend is the presence of hydrophilic groups in oleic acid. These groups repels drug molecules and reduce the entrapment efficiency. Many studies have reported variation in the entrapment efficiency of active ingredients in carriers using various lipids or oils. This is because drugs demonstrate a variable solubility in these lipids and oils [37,38].

### 3.6. Antibacterial Assay

**Figure 4:**
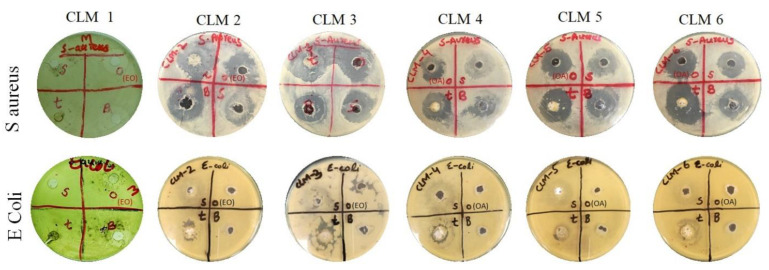
Antibacterial assays of all formulations against *S. aureus* and *E. coli* bacteria. In this figure, ‘t’ represents microbeads, ‘s’ represents the standard drug (clarithromycin), ‘o’ represents the tested oil (either eucalyptus oil (EO) or oleic acid (OA)), and ‘B’ represents the blank (0.1 N HCl).

Clarithromycin belongs to the macrolide antibiotic class and is effective against many Gram-positive and negative bacteria [10]. In this study, *S. aureus* and *E. coli* were taken as representative Gram-positive and Gram-negative bacteria, respectively. These strains were tested against the developed microbeads, the pure drug, and oils (eucalyptus oil and oleic acid) to evaluate the respective zones of inhibition. Overall, *S. aureus* was found to be more sensitive to these treatments as compared to *E. coli,* as evident from Figure 4. The probable answer lies behind the structural differences between the two strains. Gram-negative bacteria possess an additional hydrophilic lipo-polysaccharide layer, which resists the entry of hydrophobic constituents [27,29]. It has been observed that routine use of higher doses of antibiotics can lead to toxicity and the development of resistance. Pharmaceutical scientists are consistently trying to explore new and safe ways to overcome the above-mentioned hurdles to treat various infections. Therapeutic oils from natural sources possess potential antibacterial effects and can be safely combined with synthetic molecules for synergistic effects. Our results demonstrate synergistic activity between the drug and oils as evident from the higher zone of inhibition of the microbeads as compared to the pure drug and oils (Figure 4). Our results are supported by previous results where authors have reported synergistic activity between therapeutic oils and synthetic antibiotics [29,39,40,41,42]. From a technological point of view, the prepared beads showed better antimicrobial effects than clarithromycin. Similar observations have been reported previously for clarithromycin-loaded poly(d, l-lactide-co-glycolide) nanoparticles, which was attributed to the controlled release of the active ingredient and the better penetration of the nanoparticles into treated bacterial cells [43]. Based on the optimum physicochemical properties and strong antibacterial activity of CLM3 and CLM6, these two formulations were carried further for testing and analysis.

### 3.7. Scanning Electron Microscopy (SEM)

**Figure 5:**
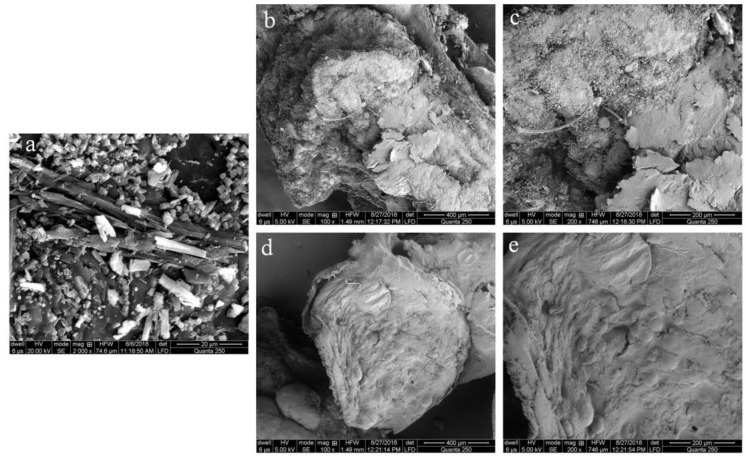
SEM microphotograph of (**a**) clarithromycin, (**b**,**c**) CLM3, and (**d**,**e**) CLM6.

SEM of the pure drug, CLM3, and CLM6 revealed detailed information about the external morphologies, as shown in Figure 5. Clarithromycin showed a crystalline nature as reported in the literature [11] and was further confirmed by XRD pattern of clarithromycin which show distinct crystalline peaks as shown in Figure 7. Under SEM, the beads appeared in a collapsed form, probably because of the removal of water during drying. Moreover, as a low concentration of polymer was used for the development of the microbeads, they have a loose network that collapsed on drying. Similar results have been reported for enalapril maleate-loaded floating particles based on sodium alginate. Particles observed to contain a low concentration of alginate collapsed after drying owing to the loose polymeric network [44]. Moreover, CLM6 microbeads showed less cracking and a comparatively smooth surface as compared to CLM3 (Figure 5). Similar results were reported by Bani-Jaber, Aideh et al. where under SEM, microbeads developed with oleic acid produced a smooth surface with few cracks [45].

### 3.8. Fourier Transform Infrared Spectroscopy

**Figure 6:**
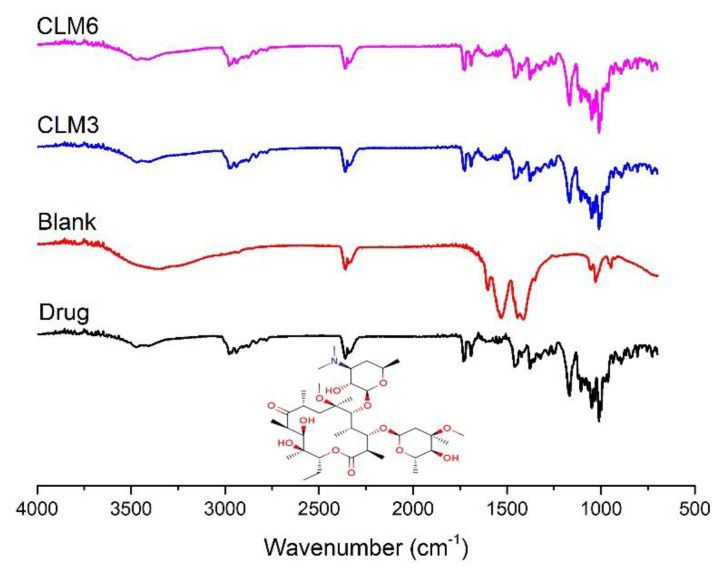
FTIR spectra of CLM3, CLM6, blank microbeads, and clarithromycin.

The FTIR spectra of clarithromycin, drug loaded, and blank hydrogel beads are shown in Figure 6. The pure drug shows an absorption band at 3471 cm^−1^, 2977 cm^−1^, 2360 cm^−1^, 2341 cm^−1^, 1691 cm^−1^, and 1731 cm^−1^ which is due to the stretching of (O–H), (C–H), (C≡N), (C≡C), carbonyl groups (C=O), and lactone rings, respectively. Moreover, the absorbance bands of oxygen (ether linkage) were recorded at 1172 cm^−1^, 1057 cm^−1^, and 1008 cm^−1^ [19,46]. The characteristic peak at 1596 cm^−1^ indicates the stretching of a (COO-) group in sodium alginate, which was observed in the blank as well as both formulations [47]. Similarly, HPMC shows the absorption peak at 1050 cm^−1^, which is due to stretching of the (C–O) group [48] and was observed in all formulations (Figure 6). The characteristic peak of the pure drug was not altered after encapsulation, indicating an absence of interaction between the drug and polymers.

### 3.9. X-ray Diffraction (XRD)

**Figure 7:**
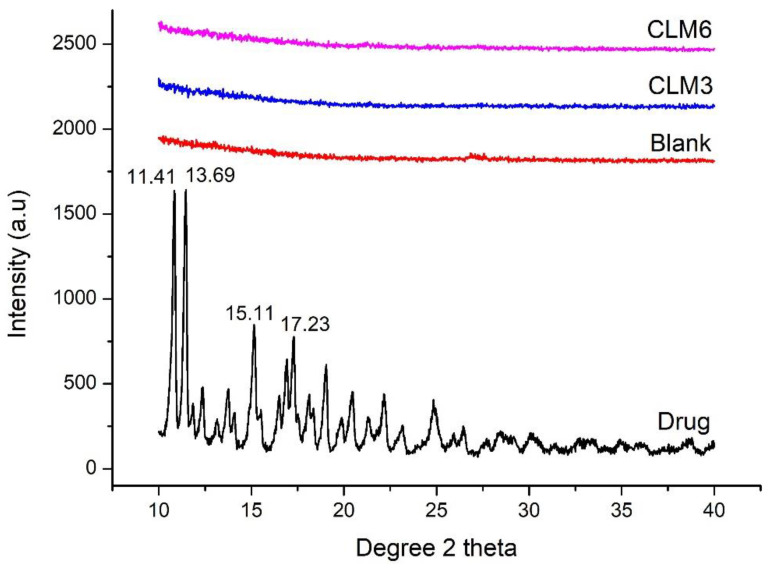
XRD diffraction pattern of CLM3, CLM6, blank microbeads, and clarithromycin.

X-ray diffraction describes the qualitative analysis of the crystalline phases and the state of crystallinity of the given material under test. The diffractograms of clarithromycin, blank, CLM3, and CLM6 are shown in Figure 7. The diffractogram of clarithromycin revealed the characteristic diffraction pattern for a crystalline material with intense reflections at Bragg’s angle (2θ) of 11.41°, 13.69°, 15.11°, 17.23°, 20.38°, and 23.08°. This confirms the crystalline nature of clarithromycin as previously described in the literature [19]. The XRD results are also supported by our SEM analysis in which crystalline drug particles are clearly visible. In contrast, the XRD diffractogram of the blank, CLM3, and CLM6 showed a diffuse pattern indicating the amorphous nature of the carrier and the distribution of the drug at molecular level (Figure 7). Similar results have been previously reported where authors observed a significant reduction in clarithromycin peaks after encapsulation in muco-adhesive alginate beads [11].

### 3.10. Differential Scanning Calorimetry (DSC)

**Figure 8:**
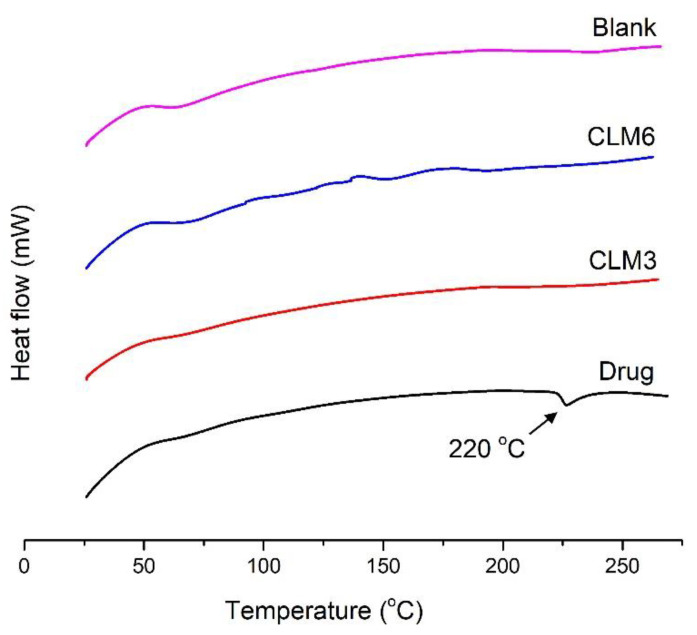
DSC thermogram of blank, CLM3 and CLM6 microbeads, and clarithromycin.

DSC analysis was conducted for clarithromycin, blank, CLM3, and CLM6 to assess their thermal behavior, as shown in Figure 8. The thermogram of the pure drug showed an endothermic peak at 220°, which corresponds to its melting point (Figure 8) and is in agreement with previously published studies about the melting point of clarithromycin [11,19]. Drug-loaded formulations did not show peaks of clarithromycin, which indicates that the formulation is stable and the drug is molecularly dispersed in the microbeads. Similar results have been reported for curcumin-loaded sodium alginate/montmorillonite polymeric composite beads, where curcumin peaks disappeared in the composite microbeads [49]. Furthermore, we did not observe degradation peaks for the blank and loaded microbeads within the test temperature and time.

### 3.11. Thermogravimetric Analysis (TGA)

**Figure 9:**
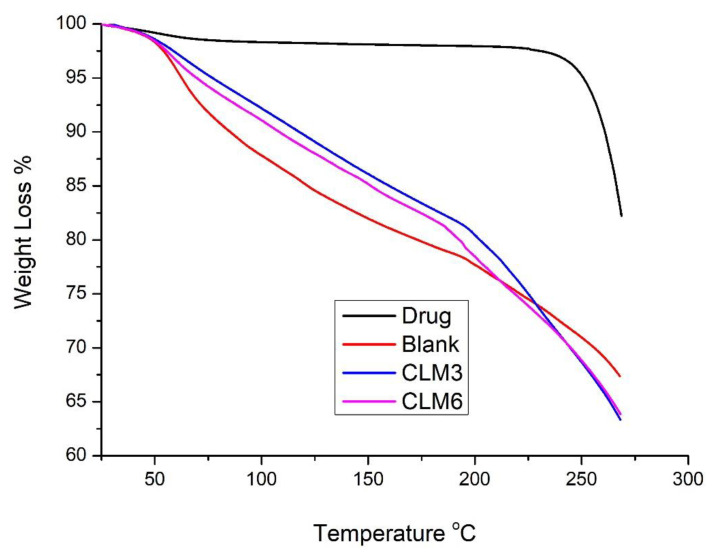
TGA graph of clarithromycin, blank, CLM3, and CLM6 formulations.

TGA was conducted on the pure drug and selected microbeads samples to assess the stability of samples by observing the mass losses of samples upon heating. The thermal characterization of clarithromycin, blank, CLM3, and CLM6 are shown in Figure 9. For pure clarithromycin, major thermal events started around 230 °C, representing the degradation of the drug above its melting point. We also observed abrupt mass loss for clarithromycin above 250 °C. Other authors have reported similar events for clarithromycin [50,51]. For blank and loaded microbeads, there was a mass loss between 50 and 200 °C, which was probably due to the loss of adsorbed water. No major degradation was observed in the tested temperature range, as the melting points of the polymers (sodium alginate and HPMC) are greater than 300 °C. Thus, we can conclude that the prepared microbeads are stable and the encapsulated active ingredients did not affect their stability. Our results are in agreement with previous results [52].

### 3.12. In Vitro Drug Release

**Figure 10:**
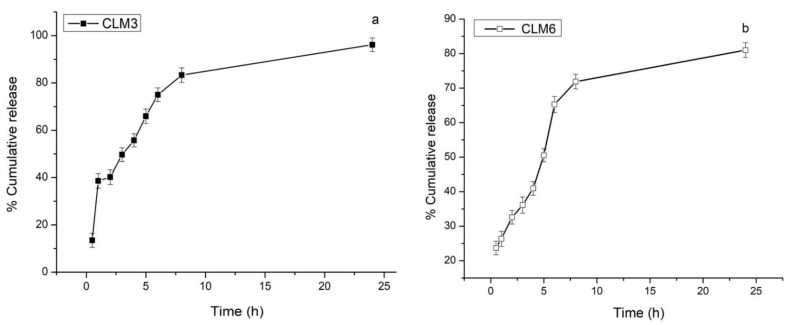
% Cumulative release of clarithromycin from (**a**) CLM3 and (**b**) CLM6.

Drug release behavior was studied for up to 24 h in simulated gastric fluid (0.1 N HCl having pH 1.2) for optimum formulation, i.e., of CLM3 and CLM6 (Figure 10). These formulations initially showed a rapid release that might be due to surface adhered drug particles or rapid release of drug present in the crona of microbeads owing to the solubility of clarithromycin in acidic pH. It is well known that during the drying of hydrogel, free drug molecules diffuse to the surface of the carrier. Upon contact with dissolution media, these molecules are rapidly released [29,53]. Afterwards, the drug is released in a sustained manner over 24 h. Similar results have been reported for clarithromycin-loaded floating microbeads that show a biphasic pattern with an initial rapid release followed by a slower release for up to six hours. After the incorporation of olive oil in microbeads, release was extended up to 12 h [11]. The controlled release of clarithromycin from microbeads in the presence of encapsulated oil could be due to the reduced porosity at the bead surface. Secondly, oil prevents rapid ingression of the dissolution media. Thirdly, HPMC K15 is a gelling agent and its presence as an interpenetrating polymer may also contribute to the slow release of the drug [11,53].

The drug release mechanism from the optimized formulations was assessed by the Korsemeyer–Peppas model (Table 4). In this model, the diffusion coefficient “n” defines the involved drug transport mechanism. If n = 0.5, the drug is released through Fickian diffusion, n > 0.5 suggests non-Fickian transport, and n = 1 describes the non-Fickian case II (zero order) transport mechanism. The correlation coefficient (R^2^) values for CLM3 and CLM6 were 0.95 and 0.99, indicating the fitness of the model used. The release exponent (n) value was 0.04 for CLM3 and 0.03 for CLM6. These values were less than 0.5, which represents Fickian diffusion [54].

## 4. Conclusions

Clarithromycin-loaded alginate floating microbeads were successfully developed to extend the stay of the carrier in the stomach by an ionotropic gelation method. Beads modified with oil (eucalyptus oil and oleic acid) provided an excellent buoyancy and drug release profile. Swelling studies were performed on all formulations to assess the impact of formulation ingredients on the swelling characteristics. The swelling of formulations containing eucalyptus oil decreases as the concentration of eucalyptus oil increases because of the lipophilic moiety in their structure, while in the case of oleic acid formulations, the swelling increases as the concentration of oleic acid increases because of the hydrophilic groups. The physical parameters of all the formulations were within an acceptable range. Clarithromycin and therapeutic oils have shown antibacterial activity against both Gram-positive (*S. aureus*) and Gram-negative (*E. coli*) bacteria, which increases with increasing concentration. The drug and oils have shown synergistic effects against representative Gram-positive and Gram-negative bacteria. Under SEM, the microbeads appeared in a collapsed form because of the desorption of water upon drying. DSC and TGA indicated a stable formulation, while FTIR confirmed the absence of an interaction between the drug and polymers. Moreover, the optimized formulation released the drug in a controlled manner over twenty-four hours. Hence, our designed microbeads could not only curtail and alleviate the shortcomings of conventional drug delivery vehicles but will also be helpful for the targeted delivery of active ingredients to the *H. pylori* infected region; further studies are needed to evaluate this aspect. Based on these findings, it is concluded that our designed microbeads have synergistic anti-bacterial activities against the tested Gram-positive and negative bacterial strains, and thus can be used in the management of a wide variety of gastric disorders. Further studies to evaluate the efficacy of these microbeads against *H. pylori* using a series of in vitro and in vivo models are therefore warranted.

## Figures and Tables

**Table 1:** Formulations of floating microbeads containing therapeutic oils and clarithromycin.

Code	Sodium Alginate (% *w*/*v*)	HPMC (% *w*/*v*)	CaCO_3_ (% *w*/*v*)	Eucalyptus Oil (% *w*/*v*)	Oleic Acid (% *w*/*v*)	Clarithromycin (mg)
CLM 1	0.5	0.25	0.5	0.3	-	120
CLM 2	0.5	0.25	0.5	0.4	-	120
CLM 3	0.5	0.25	0.5	0.5	-	120
CLM 4	0.5	0.25	0.5	-	0.20	60
CLM 5	0.5	0.25	0.5	-	0.25	60
CLM 6	0.5	0.25	0.5	-	0.30	60
CLM 7	0.5	0.25	0.5	-	-	-

**Table 2:** % Floating at different time intervals and floating lag time of all formulations.

Code	1 h	2 h	3 h	4 h	24 h	Floating Lag Time
CLM 1	60%	10%	6%	2%	-	<1 min
CLM 2	50%	24%	18%	8%	-	<1 min
CLM 3	72%	64%	64%	60%	6%	<1 min
CLM 4	52%	52%	52%	48%	-	<1 min
CLM 5	50%	48%	44%	44%	-	<1 min
CLM 6	66%	60%	40%	40%	4%	<1 min

**Table 3:** Physical characteristics of alginate microbeads.

Code	Bulk Density (g/cm^3^)	% Drug Loading	% Entrapment Efficiency
CLM 1	0.33 ± 0.0006	2.51	26.10
CLM 2	0.32 ± 0.0009	2.76	28.71
CLM 3	0.29 ± 0.001	3.09	32.24
CLM 4	0.28 ± 0.033	2.85	29.78
CLM 5	0.31 ± 0.017	2.36	24.66
CLM 6	0.26 ± 0.03	2.04	21.26

**Table 4:** In vitro clarithromycin release kinetics from optimized microbeads.

Code	Korsemeyer–Peppas Model	
	Correlation Coefficients (R^2^)	Release Exponent (n)
CLM 3	0.95	0.04
CLM 6	0.99	0.03

## Data Availability

Most of the data is presented in main article. Raw or processed data cannot be shared at this time due to technical or time limitations.

## References

[1] Naseem F., Shah S.U., Rashid S.A., Farid A., Almehmadi M., Alghamdi S. (2022). Metronidazole Based Floating Bioadhesive Drug Delivery System for Potential Eradication of *H. pylori*: Preparation and In Vitro Characterization. Polymers.

[2] Lopes C.M., Bettencourt C., Rossi A., Buttini F., Barata P. (2016). Overview on gastroretentive drug delivery systems for improving drug bioavailability. Int. J. Pharm..

[3] Kumar M., Kaushik D. (2018). An Overview on Various Approaches and Recent Patents on Gastroretentive Drug Delivery Systems. Recent Pat. Drug Deliv. Formul..

[4] Vrettos N.N., Roberts C.J. (2021). Gastroretentive Technologies in Tandem with Controlled-Release Strategies: A Potent Answer to Oral Drug Bioavailability and Patient Compliance Implications. Pharmaceutics.

[5] Klausner E.A., Lavy E., Friedman M., Hoffman A. (2003). Expandable gastroretentive dosage forms. J. Control. Release.

[6] Talukder R., Fassihi R. (2004). Gastroretentive delivery systems: A mini review. Drug Dev. Ind. Pharm..

[7] Garg R., Gupta G. (2008). Progress in controlled gastroretentive delivery systems. Trop. J. Pharm. Res..

[8] Namdev A., Jain D. (2019). Floating Drug Delivery Systems: An Emerging Trend for the Treatment of Peptic Ulcer. Curr. Drug Deliv..

[9] Choudhary S., Jain A., Amin M.C.I.M., Mishra V., Agrawal G.P., Kesharwani P. (2016). Stomach specific polymeric low density microballoons as a vector for extended delivery of rabeprazole and amoxicillin for treatment of peptic ulcer. Colloids Surf. B Biointerfaces.

[10] Israr M., Pugliese N., Farid A., Ghazanfar S., Di Cerbo A., Muzammal M., Alamri A.S., Basheeruddin Asdaq S.M., Ahmad A., Khan K.A. (2022). Preparation and Characterization of Controlled-Release Floating Bilayer Tablets of Esomeprazole and Clarithromycin. Molecules.

[11] Adebisi A.O., Laity P.R., Conway B.R. (2015). Formulation and evaluation of floating mucoadhesive alginate beads for targeting *Helicobacter pylori*. J. Pharm. Pharmacol..

[12] Savoldi A., Carrara E., Graham D.Y., Conti M., Tacconelli E. (2018). Prevalence of Antibiotic Resistance in Helicobacter pylori: A Systematic Review and Meta-analysis in World *Health Organ*. Regions. Gastroenterol..

[13] Fagoonee S., Pellicano R. (2019). *Helicobacter pylori*: Molecular basis for colonization and survival in gastric environment and resistance to antibiotics. A short review. Infect Dis..

[14] Iglesias N., Galbis E. (2020). In-Depth Study into Polymeric Materials in Low-Density Gastroretentive Formulations. Pharmaceutics.

[15] Mandal U.K., Chatterjee B., Senjoti F.G. (2016). Gastro-retentive drug delivery systems and their in vivo success: A recent update. Asian J. Pharm. Sci..

[16] Saporito F., Sandri G., Bonferoni M.C., Rossi S., Boselli C., Cornaglia A.I., Mannucci B., Grisoli P., Vigani B., Ferrari F. (2018). Essential oil-loaded lipid nanoparticles for wound healing. Int. J. Nanomed..

[17] Rajinikanth P.S., Mishra B. (2009). Stomach-site specific drug delivery system of clarithromycin for eradication of *Helicobacter pylori*. Chem. Pharm. Bull..

[18] Bai Y.-X., Li Y.F. (2006). Preparation and characterization of crosslinked porous cellulose beads. Carbohydr. Polym..

[19] El Nashar N.F., Donia A.A., Mady O.Y., El Maghraby G.M. (2017). Formulation of clarithromycin floating microspheres for eradication of Helicobacter pylori. J. Drug Deliv. Sci. Technol..

[20] Rajbhar P., Sahu A.K., Gautam S.S., Prasad R.K., Singh V., Nair S.K. (2016). Formulation and Evaluation of Clarithromycin Co-Crystals Tablets Dosage Forms to Enhance the Bioavailability. Pharma Innov..

[21] Morakul B., Suksiriworapong J., Chomnawang M.T., Langguth P., Junyaprasert V.B. (2014). Dissolution enhancement and in vitro performance of clarithromycin nanocrystals produced by precipitation–lyophilization–homogenization method. Eur. J. Pharm. Biopharm..

[22] Peppas N. (1985). Analysis of Fickian and non-Fickian drug release from polymers. Pharm. Acta Helv..

[23] Amsden B., Goosen M. (1997). An examination of factors affecting the size, distribution and release characteristics of polymer microbeads made using electrostatics. J. Control. Release.

[24] Hadley D.J., Campbell K.T., Gabriel M.H., Silva E.A. (2019). Open-source 3D printed air-jet for generating monodispersed alginate microhydrogels. bioRxiv.

[25] Nama M., Gonugunta C.S.R., Veerareddy P.R. (2008). Formulation and evaluation of gastroretentive dosage forms of clarithromycin. AAPS PharmSciTech.

[26] Azad A.K., Al-Mahmood S.M.A., Chatterjee B., Wan Sulaiman W.M.A., Elsayed T.M., Doolaanea A.A. (2020). Encapsulation of black seed oil in alginate beads as a ph-sensitive carrier for intestine-targeted drug delivery: In vitro, in vivo and ex vivo study. Pharmaceutics.

[27] Marzoug H.N.B., Romdhane M., Lebrihi A., Mathieu F., Couderc F., Abderraba M., Khouja M.L., Bouajila J. (2011). Eucalyptus oleosa essential oils: Chemical composition and antimicrobial and antioxidant activities of the oils from different plant parts (stems, leaves, flowers and fruits). Molecules.

[28] Li A., Khan I.N., Khan I.U., Yousaf A.M., Shahzad Y. (2021). Gellan Gum-Based Bilayer Mucoadhesive Films Loaded with Moxifloxacin Hydrochloride and Clove Oil for Possible Treatment of Periodontitis. Drug Des. Dev. Ther..

[29] Mahmood H., Khan I.U., Asif M., Khan R.U., Asghar S., Khalid I., Khalid S.H., Irfan M., Rehman F. (2021). Shahzad, Y.; et al. In vitro and in vivo evaluation of gellan gum hydrogel films: Assessing the co impact of therapeutic oils and ofloxacin on wound healing. Int. J. Biol. Macromol..

[30] Wang Q., Geil P., Padua G. (2004). Role of hydrophilic and hydrophobic interactions in structure development of zein films. J. Polym. Environ..

[31] Reddy O.S., Subha M.C.S., Jithendra T., Madhavi C., Rao K.C. (2020). Fabrication and characterization of smart karaya gum/sodium alginate semi-IPN microbeads for controlled release of D-penicillamine drug. Polym. Polym. Compos..

[32] Patel H., Srinatha A., Sridhar B.K. (2014). External Cross-linked Mucoadhesive Microbeads for Prolonged Drug Release: Development and In vitro Characterization. Indian J. Pharm. Sci..

[33] Azad A.K., Al-Mahmood S.M.A., Kennedy J.F., Chatterjee B., Bera H. (2021). Electro-hydrodynamic assisted synthesis of lecithin-stabilized peppermint oil-loaded alginate microbeads for intestinal drug delivery. Int. J. Biol. Macromol..

[34] Sriamornsak P., Thirawong N., Puttipipatkhachorn S. (2004). Morphology and buoyancy of oil-entrapped calcium pectinate gel beads. AAPS J..

[35] Hardikar S., Bhosale A. (2018). Formulation and evaluation of gastro retentive tablets of clarithromycin prepared by using novel polymer blend. Bull. Fac. Pharm. Cairo Univ..

[36] Stops F., Fell J.T., Collett J.H., Martini L.G. (2008). Floating dosage forms to prolong gastro-retention—The characterisation of calcium alginate beads. Int. J. Pharm..

[37] Elhesaisy N. (2020). l Swidan, S. Trazodone loaded lipid core poly (ε-caprolactone) nanocapsules: Development, characterization and in vivo antidepressant effect evaluation. Sci. Rep..

[38] Basso J., Mendes M., Cova T., Sousa J., Pais A., Fortuna A., Vitorino R., Vitorino C. (2022). A Stepwise Framework for the Systematic Development of Lipid Nanoparticles. Biomolecules.

[39] Rosato A., Vitali C., De Laurentis N., Armenise D., Milillo M.A. (2007). Antibacterial effect of some essential oils administered alone or in combination with Norfloxacin. Phytomedicine.

[40] Malik T., Singh P., Pant S., Chauhan N., Lohani H. (2011). Potentiation of antimicrobial activity of ciprofloxacin by Pelargonium graveolens essential oil against selected uropathogens. Phytother. Res..

[41] Duarte A., Ferreira S., Silva F., Domingues F.C. (2012). Synergistic activity of coriander oil and conventional antibiotics against *Acinetobacter baumannii*. Phytomedicine.

[42] Lahmar A., Bedoui A., Mokdad-Bzeouich I., Dhaouifi Z., Kalboussi Z., Cheraif I., Ghedira K., Chekir-Ghedira L. (2017). Reversal of resistance in bacteria underlies synergistic effect of essential oils with conventional antibiotics. Microb. Pathog..

[43] Öztürk A.A., Yenilmez E., Özarda M.G. (2019). Clarithromycin-Loaded Poly (Lactic-co-glycolic Acid) (PLGA) Nanoparticles for Oral Administration: Effect of Polymer Molecular Weight and Surface Modification with Chitosan on Formulation, Nanoparticle Characterization and Antibacterial Effects. Polymers.

[44] Abbas A.K., Alhamdany A.T. (2020). Floating Microspheres of Enalapril Maleate as a Developed Controlled Release Dosage Form: Investigation of the Effect of an Ionotropic Gelation Technique. Turk. J. Pharm. Sci..

[45] Bani-Jaber A., Aideh K., Hamdan I., Maraqa R. (2009). Drug-loaded casein beads: Influence of different metal-types as cross-linkers and oleic acid as a plasticizer on some properties of the beads. J. Drug Deliv. Sci. Technol..

[46] Gattani S.G., Savaliya P.J., Belgamwar V.S. (2010). Floating-mucoadhesive beads of clarithromycin for the treatment of *Helicobacter pylori* infection. Chem. Pharm. Bull..

[47] Rasel M.A.T., Hasan M. (2012). Formulation and evaluation of floating alginate beads of diclofenac sodium. Dhaka Univ. J. Pharm. Sci..

[48] Van der Weerd J., Kazarian S.G. (2005). Release of poorly soluble drugs from HPMC tablets studied by FTIR imaging and flow-through dissolution tests. J. Pharm. Sci..

[49] Reddy O.S., Subha M.C.S., Jithendra T., Madhavi C., Rao K.C. (2021). Curcumin encapsulated dual cross linked sodium alginate/montmorillonite polymeric composite beads for controlled drug delivery. J. Pharm. Anal..

[50] Chung D., Song Y.G., Kang I.M., Choi W., Park C., Song Y. (2018). Development and characterization of clarithromycin-smectite hybrid for burst release at high pH condition. Mater. Res. Express.

[51] Ijaz Q.A., Latif S., Rashid M., Arshad M.S., Hussain A., Bukhari N.I., Riaz S., Abbas N. (2021). Preparation and Characterization of pH-Independent Sustained-Release Tablets Containing Hot Melt Extruded Solid Dispersions of Clarithromycin. AAPS PharmSciTech.

[52] Balanč B., Kalušević A., Drvenica I., Coelho M.T., Djordjević V., Alves V.D., Sousa I., Moldão-Martins M., Rakić V., Nedović V. (2016). Calcium–alginate–inulin microbeads as carriers for aqueous carqueja extract. J. Food Sci..

[53] Patel N., Lalwani D., Gollmer S., Injeti E., Sari Y., Nesamony J. (2016). Development and evaluation of a calcium alginate based oral ceftriaxone sodium formulation. Prog. Biomater..

[54] Tripathi G., Singh S. (2010). Formulation and In Vitro Evaluation of pH-sensitive oil-entrapped buoyant beads of Clarithromycin. Trop. J. Pharm. Res..

